# Supplementary material for: Acceptance and compliance with micronutrient powder (MNP) among children aged 6–23 months in northern Nigeria
Source: PLOS Glob Public Health. 2022 Oct 17;2(10):e0000961. doi: 10.1371/journal.pgph.0000961 (PMC10022258; doi:10.1371/journal.pgph.0000961)
Supplement: S1 Dataset — (ZIP) [file pgph.0000961.s008.zip › S8_Dataset/Direct Observations.zip!/Direct Observations/190615_K_BK_SA_1_DO.pdf]

1.1.1 Instrument F –Direct Observation Form

**<<Instrument F Direct Observation Form>>**
**PI Name:** PragyaMathema,Stephen Kodish<br>**Study Title:** Formative research to develop an infant and young child feeding (IYCF) intervention with micronutrient powder (MNP) for children aged 6 – 23 months<br>**PI Version/Date:** Version1.0/January 24, 2015

Date: 19/06/2015 Location: Nufawa

Data Collector: Samira Adamu

Main goals of this direct observation are to answer the following questions:<br><br>1. In this household, what would be the best way(s) to give nutritional supplements to young children? Why?<br><br>2. In this household, describe the food sharing that you observe throughout the meal. Are there any predictable patterns?<br><br>3. In this household, who is favored and how is this favoritism expressed during meal preparation and eating?

Household Composition Table (Fill out as you do data collection)

INITIALS	SEX (M/F)	AGE (estimated)	Relationship to household	Description (not necessary)
BGB	F	20months	Youngest child	Baby girl with black rope tied to the neck
M	F	17years	Mother	Mother of the baby with blue and yellow wrapper
ML	F	45years	Mother-in-law	Black woman with blue and white scarf
FL	M	52years	Father-in-law	Tall dark man with light blue clothes
BGG	F	6years	Cousin	Girl wearing a green wrapper with grey veil
BBC	M	3years,6months	Cousin	Dark boy wearing a yellow top

F	M	25 years	father	Tall and dark wearing orange clothes
BG	F	15months	neighbor	Baby girl, dark in complexion from the neighborhood

PARTICIPANT ID: _________

TIME	ACTOR INITIALS	RECIPIENT INITIALS	WHAT HAPPENED	FOOD	Reactivity
10:05	M	BGB	Rubbing tropical ointment on the child while sleeping		Started using tropical ointment on her rashes
10:10	BGB		Sleeping on the mat		
10:15	BGB		Sleeping on the mat		
10:15	M		Washing clothes in the surrounding		
10:15	ML		Giving the animals, animal feeds		
10:20	M		Trying to make the baby up i.e rubbing powder, eyeliner and lipstick		R
10:25	BGB		Still sleeping on the mat		
10:25	M	BGB	Wearing pants for the baby		R
10:30	M		Washing clothes		
	BGB		Sleeping on the mat		

TIME	ACTOR INITIALS	RECIPIENT INITIALS	WHAT HAPPENED	FOOD	Reactivity
10:35	BGB		Sleeping on the mat		
	M		Spreading mat on the floor		
	ML		Lying down under the tree		
10:40	BGB		Sleeping on the mat		
10:45	BGB		Woke up and started crying		
	M		Still sleeping		
	ML		Lying under the tree		
10:50	M	BGB	Feeding the baby kunu	Kunun millet	
10:55	M	BGB	Feeding the baby kunu	Kunun millet	
11:00	BGB		Sitting on the mat		
	M	BGB	Wearing clothes for the baby		
11:05	ML	BGB	Carrying the child		
	M		Washing clothed		
11:10	ML	BGB	Carrying the child		
11:15	BGB		Sitting on the stool at grand mother’s side		
	M		Still washing clothes		
	ML		Carrying a bucket of water to the toilet		
11:20	M		Drying clothes on the fence		

TIME	ACTOR INITIALS	RECIPIENT INITIALS	WHAT HAPPENED	FOOD	Reactivity
	BGB		Still sitting on the stool		
11:25	BGB		Crying and holding mother's leg		
	M		Still washing clothes		
11:30	ML	BGB	Carrying the baby outside to another household		
11:35	ML	BGB	Backing the baby and trekking to another household		
11:40	ML	BGB	Backing the baby and trekking to another household		
11:45	ML	BGB	Backing the baby and trekking to another household		
11:50	ML	BGB	Backing the baby and greeting the household persons<br>A naming ceremony is going to take place		
11:55	BGB		Playing beside the grandmother.<br>Some women are frying waina (rice puff puff)		
12:00	BGB	BGB	Eating waina by herself	Waina gyero	
		ML	frying waina	Waina gyero	
12:05	BGB	BGB	Feeding herself waina	Waina gyero	
	ML		Still frying waina		
12:10	BGB	BGB	Feeding herself waina	Waina gyero	

TIME	ACTOR INITIALS	RECIPIENT INITIALS	WHAT HAPPENED	FOOD	Reactivity
12:15	BGB	BGB	Feeding herself waina	Waina gyero	
12:20	BGB		Carrying a black polythene back with waina inside	Waina gyero	
	ML		Standing beside the child		
12:25	BGG	BGB	Backing the child		
	BGB		Carrying the black polythene with waina	Waina gyero	
12:30	BGG	BGB	Backing the child		
	BGB		Holding the black polythene containing waina	Waina gyero	
12:35	BGG	BGB & BBL	Backing the baby and holding boys and trekking back to the household		
12:40	BGG	BGB & BBL	Backing the baby and holding boys and trekking back to the household		
12:45	BGG		Sharing the waina to the child and other son	Waina gyero	
	M		Sieving grinded watery millet to remove shaft	Millet	
12:50	BGB	BGB	Still eating waina	Waina millet	
	M		Still sieving grinded watery millet to remove shaft	Millet	
12:55	BGB	BGB	Still eating waina and playing with slippers	Waina millet	

TIME	ACTOR INITIALS	RECIPIENT INITIALS	WHAT HAPPENED	FOOD	Reactivity
1:00	BGB		Stooling on the floor		
1:05	M	BGB	Cleaning and washing the child		
1:10	BGB		Playing with cousins		
1:15	BGB		Playing with sand		
1:15	M		Lying beside her grandmother		
1:20	BGB		Playing with sand		
1:25	BGB		Playing with sand		
1:30	BGB		Still playing with sand		
	M		lying down beside her		
1:35			The baby is crying		
1:40	BGB	BGB	Eating dabino		
1:45	BGB		The baby is crying		
	F		Holding the baby's hand to the room		
2:00	BGB		The baby is still crying		
	M	BGB	Carrying the baby on her laps		
		BGB	The baby is sleeping		
2:05		M	She is tying the goats		
2:10	BGB		She is sleeping		
2:15	BGB		She is sleeping		
	M		Giving the animals feed		
2:20	BGB		She is sleeping		

TIME	ACTOR INITIALS	RECIPIENT INITIALS	WHAT HAPPENED	FOOD	Reactivity
2:25	BGB		She is sleeping		
2:30	BGB		She is sleeping		
2:35	BGB		She is sleeping		
2:40	BGB		She is sleeping		
	FL		Coming back from the mosque		
2:45	BGB		She is sleeping		
2:50	BGB		She is sleeping		
2:55	BGB		She is sleeping		
	M		She is washing plates and bowls		
3:00	BGB		She is sleeping		
	M		She is washing plates and bowls		
3:05	BGB		She is sleeping		
	M		She is washing plates and bowls		
3:10	BGB		She is sleeping		
	M		She is washing plates and bowls		
3:15	BGB		She is sleeping		
3:20	BGB		She is sleeping		
3:25	BGB		She is sleeping		
3:30	BGB		She is sleeping		
	BGB		She is sleeping		

TIME	ACTOR INITIALS	RECIPIENT INITIALS	WHAT HAPPENED	FOOD	Reactivity
3:35	BGB	BGB	She is eating dakaule	moringa	
3:40	BGB	BGB	She is drinking kunu	Kunun millet	
3:45	BGB	BGB	She is drinking kunu	Kunun millet	
3:50	BGB	BGB	She is drinking kunu	Kunun millet	
3:55	BGB		She is crying		
4:00	M	BGB	She is bathing the baby		
4:05	M	BGB	She is bathing the baby		
4:10	BGB		Playing in water		
4:15	BGB		Playing in water		
	ML		Coming into the household		
4:20	BGB		Happily playing with grandmother		
4:25	BGB		Playing alone with sand		
4:30			Playing beside the mother		
4:35			She is praying		
4:40	BGB		Playing around alone		
4:45	BGB		Playing around alone		
	ML		Observing prayers		
4:50	BGB		Roaming about alone		

4:55	BGB		Playing and holding mother's leg		
	M		Walking and playing with child		
5:00	BGB		Lying on the mat		
	ML		Lying with the child on the mat		
5:05	BGB		Playing with empty carton		
5:10	BGB		Playing in the neighborhood with two other children		
5:15	BGB&BG		Playing with child from the neighborhood		
5:20	BGB		Playing with chicken in then house		
	F		Fixing standing fan		
5:25	BGB		Playing with other children from the neighborhood		
5:30	BGB		Roaming around in the house		
5:35	BGB	BGB	Feeding with kunu	kunu millet	
5:40	BGB		Feeding with kunu	Kunu millet	
5:45	BGB&BG		Playing together with sand		
5:50	BGB&BG		Playing together in the neighborhood		
5:55	ML		Sharing waina to neighbor's children		
	ML	BGB	Giving the baby waina	Waina maize	
6:00	BGB	BGB	Eating waina with miyan kuka		
6:05	M		Setting fire and putting pot on fire		

	ML		Fetching water from well		
	M&ML		Both sieving well water into pot		
	BGB	BGB	Eating waina and miyan kuka		
6:10	M		Setting fire		
	M	BGG	Adding more waina for the baby	Waina maize	
	BGB		Eating waina		
			Still fetching water from the well		
6:15	ML/BGB		Came back from fetching water		
6:20	M	BGB	Carrying the baby on her laps		
6:25	M	BGB	Backing the baby because e of the wind		
6:30	M	BGB	Backing the baby and packing things from outside because it may rain because its cloudy<br>Pot is still on fire		
6:35	ML		Pounding kuli-kuli		
	ML	BGB	Giving the child kuli-kuli		
6:40	ML		Preparing paste from sieved millet, though it was raining heavily		
	ML		Fetching small portion of the paste on the small bowl	Millet	
	ML		Adding boiled water into the large quantity of paste using a sieve to sieve the water off particles		
	ML		Stirring the kunu	Kunun millet	

	ML		Adding more boiling water to the kunu		
	ML		Carrying the kunu to her room	Kunun millet	
	ML		Stirring the kunu again	Kunun millet	
6:50	M	BGB	Backing the baby		
6:53	ML		Adding then small portion fetched from the paste to make the kunu thicker	Kunun millet	
6:55	ML		Adding more of the small portion fetched to the kunu to make it thick		
6:55	M	BGB	Carrying the child her laps and covering her		
7:00	M	BGB	Carrying the child her laps and covering her		
7:05	M	BGB	Carrying the child her laps and covering her		
7:10	M	BGB	Giving the child double dose of multi vitamin because the child missed the afternoon dose		
7:15	ML		Brought kunu for the son and daughter in-law	Kunun millet	
7:20	M	BGB	Backing the baby		
	F		Preparing a mango juice with sachet water and cow milk		
	F	M	Giving the juice prepared		
7:25	BGB		Taking orange juice		

	F	BGB	Giving her kwai da kwai bu the BGB refused to collect		
7:30	F	BGB	Giving her orange juice		
	BGB	BGB	Eating the kwaik-kwai	Soya bean cake	
7:35	ML		Bringing fanta, orange and bread for me		
	BGB		Still eating kwai-da-kwai	Soya bean cake	
7:40	M	BGB	Backing the baby to perform ablution		
7:45	F		Bought rice (jollof) for me		
	M	BGB	Gave some portion of the rice to the child		
7:50	BGB	BGB	Eating the rice		
7:50	M	F	Serving the husband kunu and kosai		
	ML	FL			
7:55	BGB	BGB	Eating jollof rice		
8:00	F	BGB	Carrying the baby on his lap		

Primary Questions (Complete <u>after</u> you complete data collection):

From what you have seen observing THIS HOUSEHOLD, please answer the following questions in detail:

Question	Write your <u>detailed</u> response here
1. In this household, what would be the best way(s) to give nutritional supplements such as an MNP to young children to ensure compliance?<br><br>Why?	The best way to give the MNP is through the mother and mother-in-law (grandmother).<br>Also the father because he will ensure proper usage without over dosage<br><br>Because the child is taken care of by both mother and grandmother.<br>She is so close to both of them that she cries when any of them leave her side.
2. In this household, who is favored during meal preparation or feeding?<br><br>How is this favoritism expressed?	The child is the most favored during feeding. The mother is also favored.<br><br>Because she is served the meal first before any of the parent eats and she is being watched to eat before they eat.<br>The mother is given different types of food when she is pregnant.
3. How could we promote the MNP supplement for this family that would attract their attention?<br><br>Who would they listen to?	The MNP supplement can be promoted by sensitizing both the father and the father-in-law (grandfather) and create good awareness of the product.<br><br>The mother and grandmother will listen to the father and grandfather because women of this area are all answerable to their husbands and father-in-laws.<br>Also, then father and grandfathers will listen to the community

	leaders because he stands for the law in the community.
4. In this household, what would be the best ways to promote the MNP but limit how much it would be shared?<br><br>Why?	There is little or no sharing in the household because the child is alone, although she is being visited by cousins and neighbors occasionally.<br>It should be emphasized to mothers that it shouldn't be shared among other children and it should be considered as medicine maybe.
5. What other issues and concerns did you see with this family that is important for us to consider with regard to introduction of an MNP?<br><br>Why?	Lack of food, because the child was fed with kunu millet throughout the day and even the household visited, she was offered waina millet.<br><br>Lack of proper hygiene; although the mother is trying to clean up but no proper measures was taken when the child was stooling and the farm animals were also drinking and eating from cooking pots and bowls.

6. Based on your experience observing this child today, do you believe reactivity negatively impacted your ability to see the food-related behaviors of the child?<br><br>Please explain your answer in detail.	No, it did not affect the work negatively.<br><br>Because the child exhibits her mode and pattern of eating behavior without any obstruction.
